# Crystal structure and Hirshfeld surface analysis of 4-allyl-6-bromo-2-(4-chloro­phen­yl)-4*H*-imidazo[4,5-*b*]pyridine

**DOI:** 10.1107/S2056989018017322

**Published:** 2019-01-01

**Authors:** Selma Bourichi, Youssef Kandri Rodi, Tuncer Hökelek, Amal Haoudi, Catherine Renard, Frédéric Capet

**Affiliations:** a Laboratoire de Chimie Organique Appliquée, Université Sidi Mohamed Ben Abdallah, Faculté des Sciences et Techniques, Route d’immouzzer, BP 2202, Fez, Morocco; b Department of Physics, Hacettepe University, 06800 Beytepe, Ankara, Turkey; c Unité de Catalyse et de Chimie du Solide (UCCS), UMR 8181, Ecole Nationale Supérieure de Chimie de Lille, Université Lille 1, 59650 Villeneuve d’Ascq Cedex, France

**Keywords:** crystal structure, imidazo[4,5-*b*]pyridine, Hirshfeld surface

## Abstract

The title compound, C_15_H_11_BrClN_3_, is built up from a planar imidazo[4,5-*b*]pyridine unit linked to phenyl and allyl substituents. The allyl substituent is rotated significantly out of the imidazo[4,5-*b*]pyridine plane, while the benzene ring is inclined by 3.84 (6)° to the ring system. In the crystal, mol­ecules are linked *via* a pair of weak inter­molecular C—H⋯N hydrogen bonds, forming an inversion dimer with an *R*_2_^2^(20) ring motif. The dimers are further connected by π–π stacking inter­actions between the imidazo[4,5-*b*]pyridine ring systems [centroid–centroid distances = 3.7161 (13) and 3.8478 (13) Å]. The important contributions to the Hirshfeld surface are H⋯H (35.9%), H⋯Cl/Cl⋯H (15.0%), H⋯C/C⋯H (12.4%), H⋯Br/Br⋯H (10.8%), H⋯N/N⋯H (7.5%), C⋯Br/Br⋯C (5.9%), C⋯C (5.5%) and C⋯N/N⋯C (4.0%) contacts.

## Abstract 2

The imidazo[4,5-*b*]pyridine unit is planar, while the phenyl and allyl substituents are rotated a little out of this plane. In the crystal, mol­ecules are linked *via* pairs of the weak inter­molecular C—H⋯N hydrogen bonds, forming inversion dimers with 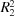
(20) ring motifs. The dimers are further connected by π–π stacking inter­actions between the imidazo[4,5-*b*]pyridine ring systems.

## Chemical context   

Heterocyclic ring systems having an imidazo[4,5-*b*]pyridine unit can be considered as structural analogues of purines and have shown diverse biological activity depending on the substituents of the heterocyclic ring. Their activities include anti­cancer (Zhiqiang *et al.*, 2005 ▸), tuberculostatic (Bukowski & Janowiec, 1989 ▸) and anti­mitotic (Parthiban *et al.*, 2006 ▸) actions. Some imidazo[4,5-*b*]pyridine derivatives have also been reported as corrosion inhibitors for steel in acidic medium (Bouayad *et al.*, 2018 ▸; Sikine *et al.*, 2016 ▸), and some of them can be used to treat peptic ulcers, diabetes and mental illness (Scribner *et al.*, 2007 ▸; Liang *et al.*, 2007 ▸).
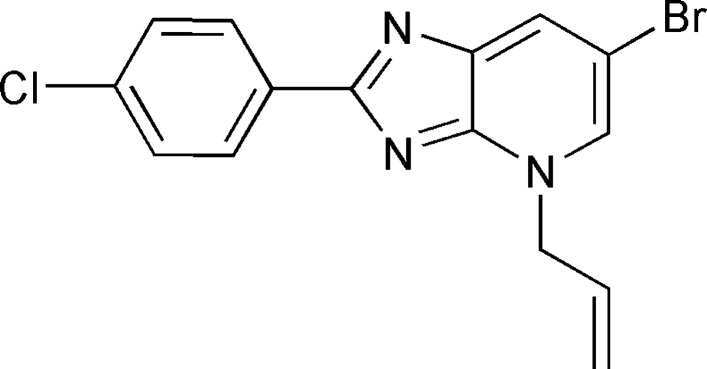


As a continuation of our research work devoted to the development of substituted imidazo[4,5-*b*]pyridine derivatives (Bourichi *et al.*, 2016 ▸; Ouzidan *et al.*, 2010*a* ▸,*b* ▸,*c* ▸), we report herein the synthesis, the mol­ecular and crystal structures along with the Hirshfeld surface analysis of the title compound, a new imidazo[4,5-*b*]pyridine derivative, which was obtained by the reaction of allyl bromide with 6-bromo-2-(4-chloro­phen­yl)-4*H*-imidazo[4,5-*b*]pyridine in the presence of a catalytic qu­antity of tetra-*n*-butyl­ammonium bromide under mild conditions.

## Structural commentary   

**Figure 1:**
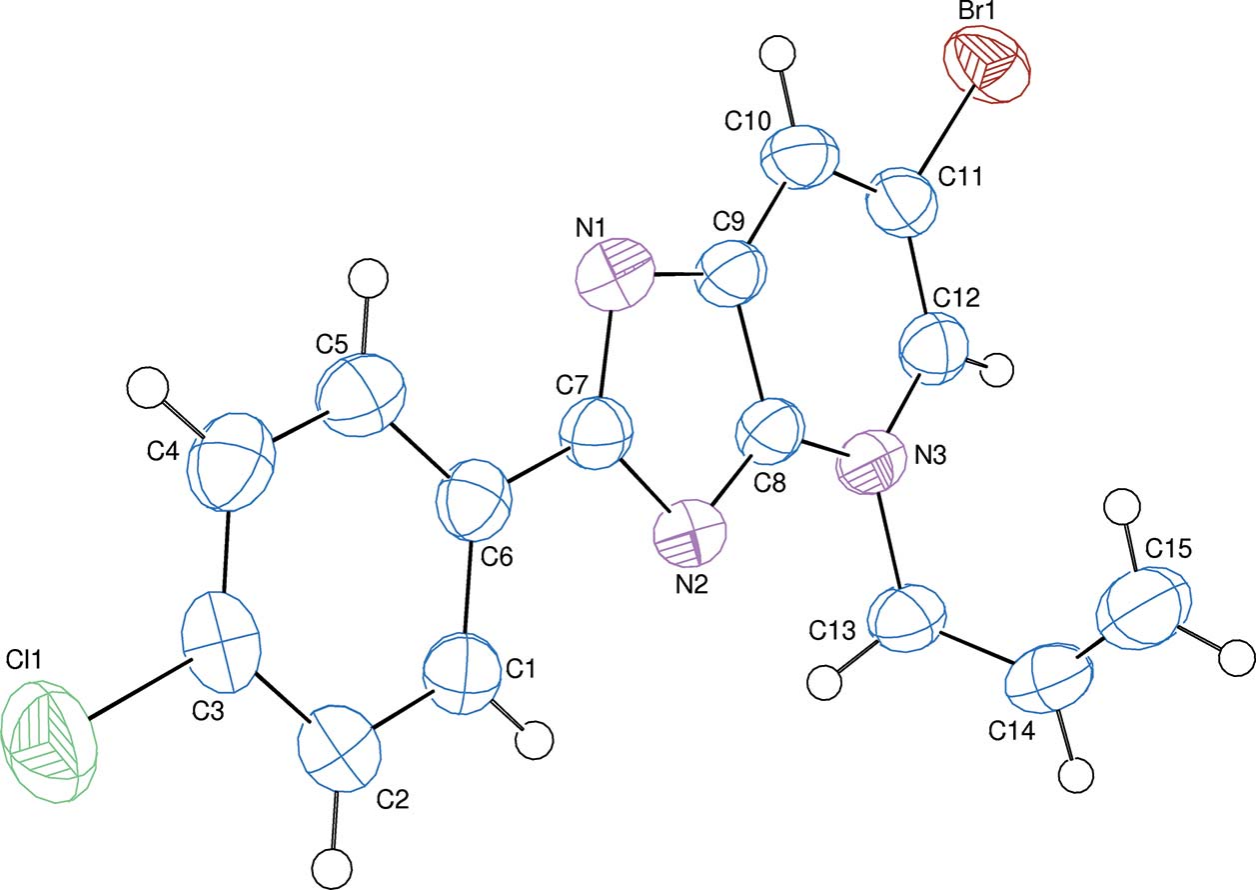
The mol­ecular structure of the title compound, showing the atom-numbering scheme. Displacement ellipsoids are drawn at the 50% probability level.

The title compound is built up from an imidazo[4,5-*b*]pyridine unit linked to phenyl and allyl substituents (Fig. 1 ▸). The imidazo[4,5-*b*]pyridine ring system is planar, with a maximum deviation of 0.016 (2) Å for atom C12. The ring system is inclined by 3.84 (6)° to the benzene C1–C6 ring, with the N2—C7—C6—C1 torsion angle being 3.3 (3)°. The allyl substituent is nearly perpendicular to the imidazo[4,5-*b*]pyridine plane, as indicated by the C8—N3—C13—C14 torsion angle of −97.3 (2)°. Atoms C6 and C13 are 0.038 (2) and 0.014 (2) Å, respectively, away from the imidazo[4,5-*b*]pyridine plane.

## Supra­molecular features   

**Figure 2:**
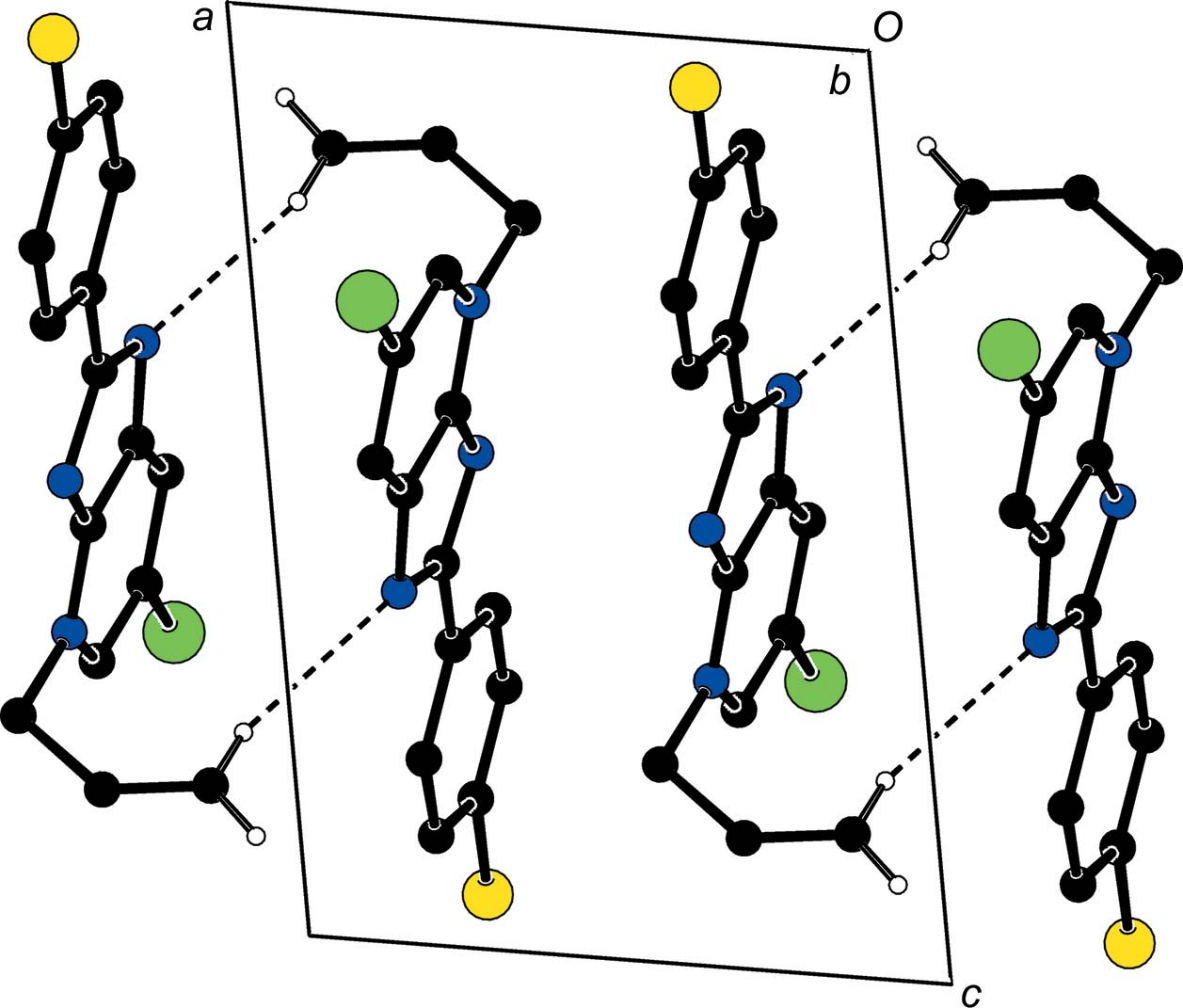
A part of the packing diagram of the title compound, viewed down [010]. The weak inter­molecular C—H⋯N hydrogen bonds are shown as dashed lines. H atoms not involved in the hydrogen bonding have been omitted for clarity.

In the crystal, mol­ecules are linked *via* a pair of weak inter­molecular C—H⋯N hydrogen bonds [C15—H15*A*⋯N1^i^; symmetry code: (i) −*x*, −*y* + 1, −*z* + 1; Table 1 ▸], forming an inversion dimer with an 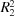
(20) ring motif (Fig. 2 ▸). The dimers are further connected by π–π stacking inter­actions between the imidazo[4,5-*b*]pyridine ring systems. The centroid–cen­troid distances, *Cg*1⋯*Cg*1^ii^ and *Cg*1⋯*Cg*2^i^ [symmetry code: (ii) −*x* + 1, −*y* + 1, −*z* + 1], are 3.7161 (13) and 3.8478 (13) Å, respectively, where *Cg*1 and *Cg*2 are the centroids of the N1/N2/C7–C9 and N3/C8–C12 rings, respectively.

## Database survey   

A non-*para*-substituated analogue, namely 4-allyl-6-bromo-2-phenyl-4*H*-imidazo[4,5-*b*]pyridine monohydrate, has been reported (Ouzidan *et al.*, 2010*c* ▸), and three similar structures, 4-benzyl-6-bromo-2-phenyl-4*H*-imidazo[4,5-*b*]pyridine (Ouzidan *et al.*, 2010*b* ▸), 4-benzyl-6-bromo-2-meth­oxy­phenyl-4*H*-imidazo[4,5-*b*]pyridine monohydrate (Ouzidan *et al.*, 2010*a* ▸) and 4-benzyl-6-bromo-2-(4-chloro­phen­yl)-4*H*-imidazo[4,5-*b*]pyridine (Bourichi *et al.*, 2017 ▸), have been also reported.

## Hirshfeld surface analysis   

**Figure 3:**
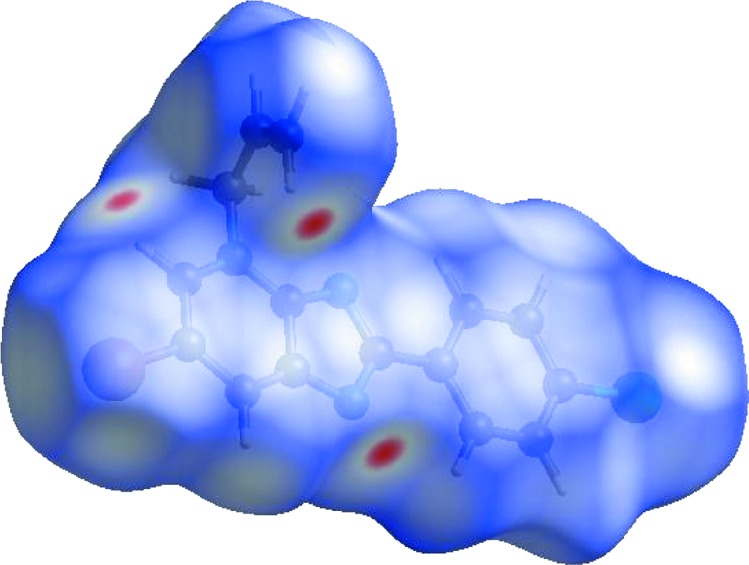
View of the three-dimensional Hirshfeld surface of the title compound plotted over *d*_norm_ in the range −0.1373 to 1.1294 a.u.

**Figure 4:**
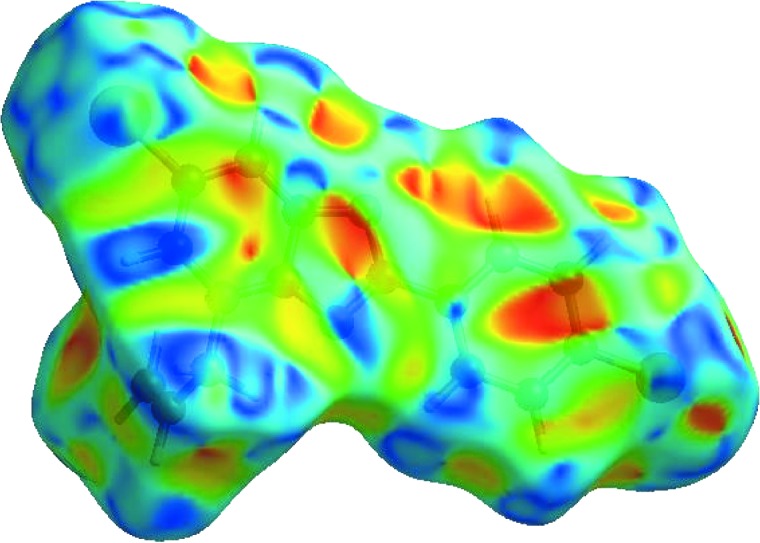
Hirshfeld surface of the title compound plotted over shape index.

**Figure 5:**
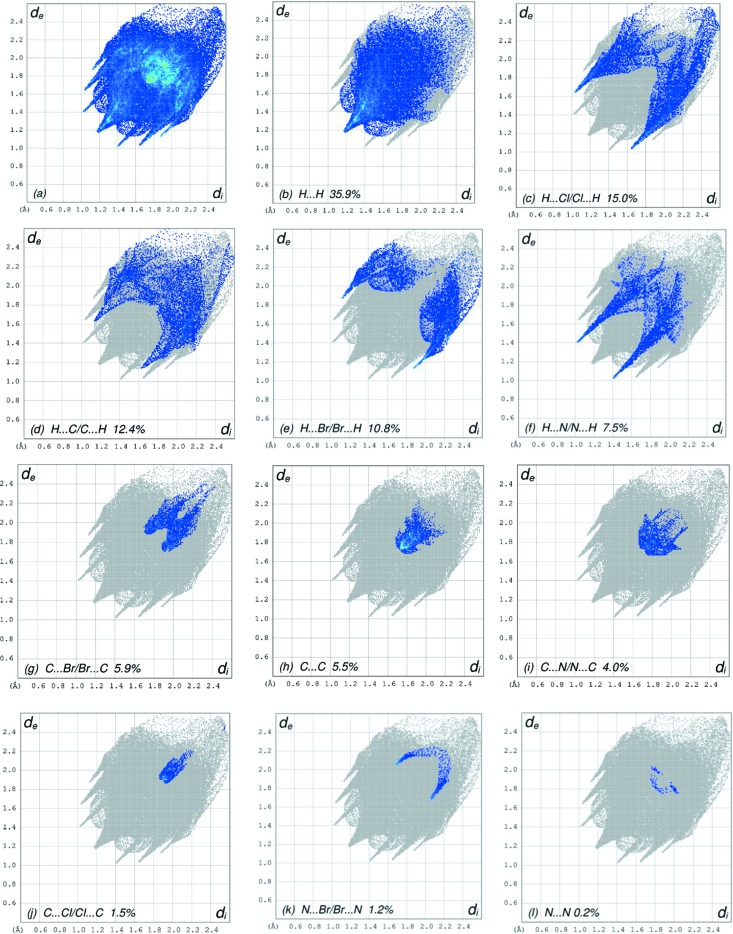
The full two-dimensional fingerprint plots for the title compound, showing (*a*) all contacts, and delineated into (*b*) H⋯H, (*c*) H⋯Cl/Cl⋯H, (*d*) H⋯C/C⋯H, (*e*) H⋯Br/Br⋯H, (*f*) H⋯N/N⋯H, (*g*) C⋯Br/Br⋯C, (*h*) C⋯C, (*i*) C⋯N/N⋯C, (*j*) C⋯Cl/Cl⋯C, (*k*) N⋯Br/Br⋯N and (*l*) N⋯N contacts. The *d*_i_ and *d*_e_ values are the closest inter­nal and external distances (in Å) from given points on the Hirshfeld surface contacts.

**Figure 6:**
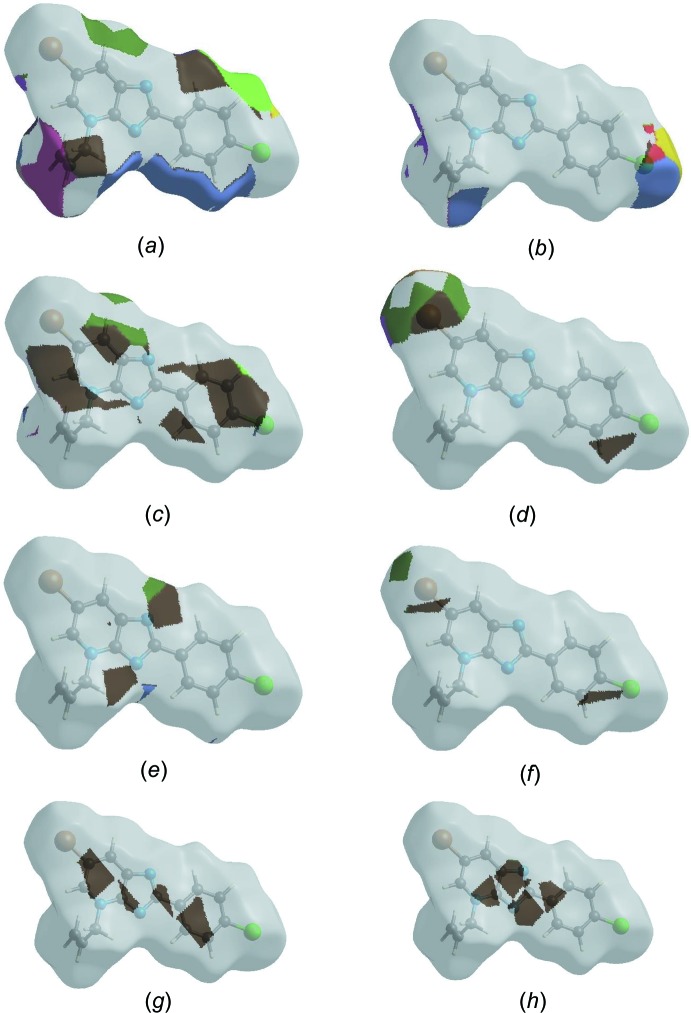
The Hirshfeld surface representations with the function *d*_norm_ plotted onto the surface for (*a*) H⋯H, (*b*) H⋯Cl/Cl⋯H, (*c*) H⋯C/C⋯H, (*d*) H⋯Br/Br⋯H, (*e*) H⋯N/N⋯H, (*f*) C⋯Br/Br⋯C, (*g*) C⋯C and (*h*) C⋯N/N⋯C contacts.

In order to visualize the inter­molecular inter­actions in the crystal of the title compound, a Hirshfeld surface (HS) analysis (Spackman & Jayatilaka, 2009 ▸) was carried out using *CrystalExplorer17.5* (Turner *et al.*, 2017 ▸). In the HS plotted over *d*_norm_ (Fig. 3 ▸), the white surface indicates contacts with distances equal to the sum of the van der Waals radii, and the red and blue colours indicate distances shorter (in close contact) or longer (distinct contact) than the sum of the van der Waals radii, respectively (Venkatesan *et al.*, 2016 ▸). The bright-red spots appearing near atoms N1 and H15*A* indicate their roles as the respective donors and/or acceptors in the dominant C—H⋯N hydrogen bond (Table 1 ▸). The shape index (Fig. 4 ▸) clearly suggests that there are π–π inter­actions, which are shown as adjacent red and blue triangles. The overall two-dimensional fingerprint plot and those delineated into H⋯H, H⋯Cl/Cl⋯H, H⋯C/C⋯H, H⋯Br/Br⋯H, H⋯N/N⋯H, C⋯Br/Br⋯C, C⋯C, C⋯ N/N⋯C, C⋯Cl/Cl⋯C, N⋯Br/Br⋯N and N⋯N contacts (McKinnon *et al.*, 2007 ▸) are illustrated in Figs. 5 ▸(*a*)–(*l*), together with their relative contributions to the Hirshfeld surface. The contributions are 35.9, 15.0, 12.4, 10.8, 7.5, 5.9, 5.5, 4.0, 1.5, 1.2 and 0.2%, respectively, for H⋯H, H⋯Cl/Cl⋯H, H⋯C/C⋯H, H⋯Br/Br⋯H, H⋯N/N⋯H, C⋯Br/Br⋯C, C⋯C, C⋯N/N⋯C, C⋯Cl/Cl⋯C, N⋯Br/Br⋯N and N⋯N contacts. The most important inter­action is H⋯H (35.9%), which is reflected as widely scattered points of high density due to the large hydrogen content of the mol­ecule [Fig. 5 ▸(*b*)]. The spike with the tip at *d*_e_ = *d*_i_ = 1.16 Å is due to the short inter­atomic H⋯H contacts. The H⋯Cl/Cl⋯H contacts (15.0%) have a nearly symmetrical distribution of points and a pair of spikes with tips at *d*_e_ + *d*_i_ = 2.67 Å [Fig. 5 ▸(*c*)]. In the absence of C—H ⋯ π inter­actions, the H⋯C/C⋯H contacts (12.4%) also have a nearly symmetrical distribution of points with tips at *d*_e_ + *d*_i_ = 2.79 Å [Fig. 5 ▸(*d*)]. The H⋯Br/Br⋯H contacts (10.8%) have a symmetrical distribution of points and a pair of spikes with tips at *d*_e_ + *d*_i_ = 3.00 Å [Fig. 5 ▸(*e*)]. A pair of spikes with tips at *d*_e_ + *d*_i_ = 2.42 Å (Fig. 5 ▸ *f*) in the H⋯N/N⋯H contacts (7.5%) arises from the C—H⋯N hydrogen bond (Table 1 ▸). The C⋯Br/Br⋯C contacts (5.9%) have a pair of wings with tips at *d*_e_ + *d*_i_ ∼ 3.62 Å [Fig. 5 ▸ *(g*)]. The C⋯C contacts (5.5%) have an arrow-shaped distribution of points with the tip at *d*_e_ = *d*_i_ = 1.75 Å [Fig. 5 ▸(*h*)]. The C⋯N/N⋯C contacts (4.0%) have wide spikes with tips at *d*_e_ + *d*_i_ = 3.44 Å [Fig. 5 ▸(*i*)]. The HS representations with the function *d*_norm_ plotted onto the surface are shown for the H⋯H, H⋯Cl/Cl⋯H, H⋯C/C⋯H, H⋯Br/Br⋯H, H⋯N/N⋯H, C⋯Br/Br⋯C, C⋯C and C⋯N/N⋯C contacts [Figs. 6 ▸(*a*)–(*h*)].

## Synthesis and crystallization   

A mixture of 6-bromo-2-(4-chloro­phen­yl)-4*H*-imidazo[4,5-*b*]pyridine (0.2 g, 0.65 mmol) dissolved in 25 ml of *N*,*N*-di­methyl­formamide (DMF) and potassium carbonate (0.13 g, 0.92 mmol) was stirred for 5 min, and then to a mixture of tetra-*n*-butyl­ammonium bromide (0.032 g, 0.1 mmol) and allyl bromide (0.094 g, 0.77 mmol) was added. Stirring was continued for 6 h at room temperature. After removing the salts by filtration, DMF was evaporated under reduced pressure, and the solid obtained was dissolved in di­chloro­methane. The residue was extracted with distilled water and the resulting mixture was chromatographed on a silica-gel column (eluent = ethyl acetate–hexane, 1:3 *v*/*v*). Brown single crystals suitable for X-ray diffraction were obtained by evaporation of an ethyl acetate–hexane (1:3 *v*/*v*) solution.

## Refinement   

Crystal data, data collection and refinement details are summarized in Table 2 ▸. All H atoms were positioned geometrically, with C—H = 0.93 or 0.97 Å, and constrained to ride on their parent C atoms, with *U*_iso_(H) = 1.2*U*_eq_(C).

## Supplementary Material

Crystal structure: contains datablock(s) I, global. DOI: 10.1107/S2056989018017322/is5505sup1.cif

Structure factors: contains datablock(s) I. DOI: 10.1107/S2056989018017322/is5505Isup2.hkl

Supporting information file. DOI: 10.1107/S2056989018017322/is5505Isup3.cdx

Supporting information file. DOI: 10.1107/S2056989018017322/is5505Isup4.cml

CCDC reference: 1883384

Additional supporting information:  crystallographic information; 3D view; checkCIF report

## Figures and Tables

**Table 1:** Hydrogen-bond geometry (Å, °)

*D*—H⋯*A*	*D*—H	H⋯*A*	*D*⋯*A*	*D*—H⋯*A*
C15—H15*A*⋯N1^i^	0.93	2.59	3.454 (4)	155

Symmetry code: (i) 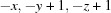.

**Table 2:** Experimental details

Crystal data	
Chemical formula	C_15_H_11_BrClN_3_
*M*_r_	348.63
Crystal system, space group	Triclinic, *P* 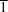
Temperature (K)	296
*a*, *b*, *c* (Å)	7.6218 (5), 8.5238 (5), 11.1093 (7)
α, β, γ (°)	95.739 (3), 98.880 (3), 94.979 (3)
*V* (Å^3^)	705.66 (8)
*Z*	2
Radiation type	Mo *K*α
μ (mm^−1^)	3.09
Crystal size (mm)	0.3 × 0.23 × 0.06

Data collection	
Diffractometer	Bruker APEXII CCD
Absorption correction	Multi-scan (*SADABS*; Bruker, 2014 ▸)
*T*_min_, *T*_max_	0.591, 0.746
No. of measured, independent and observed [*I* > 2σ(*I*)] reflections	31084, 4288, 3226
*R*_int_	0.033
(sin θ/λ)_max_ (Å^−1^)	0.715

Refinement	
*R*[*F* ^2^ > 2σ(*F* ^2^)], *wR*(*F* ^2^), *S*	0.035, 0.089, 1.03
No. of reflections	4288
No. of parameters	181
H-atom treatment	H-atom parameters constrained
Δρ_max_, Δρ_min_ (e Å^−3^)	0.57, −0.47

Computer programs: *APEX2* and *SAINT* (Bruker, 2015 ▸), *SHELXT* (Sheldrick, 2015*a* ▸), *SHELXL* (Sheldrick, 2015*b* ▸), *ORTEP-3 for Windows* and *WinGX* (Farrugia, 2012 ▸) and *PLATON* (Spek, 2015 ▸).

## supplementary crystallographic information

### Crystal data

**Table:**

C_15_H_11_BrClN_3_	*Z* = 2
*M**_r_* = 348.63	*F*(000) = 348
Triclinic, *P*1	*D*_x_ = 1.641 Mg m^−^^3^
*a* = 7.6218 (5) Å	Mo *K*α radiation, λ = 0.71073 Å
*b* = 8.5238 (5) Å	Cell parameters from 9961 reflections
*c* = 11.1093 (7) Å	θ = 2.4–25.8°
α = 95.739 (3)°	µ = 3.09 mm^−^^1^
β = 98.880 (3)°	*T* = 296 K
γ = 94.979 (3)°	Plate, colourless
*V* = 705.66 (8) Å^3^	0.3 × 0.23 × 0.06 mm

### Data collection

**Table:**

Bruker APEX-II CCD diffractometer	3226 reflections with *I* > 2σ(*I*)
φ and ω scans	*R*_int_ = 0.033
Absorption correction: multi-scan (SADABS; Bruker, 2014)	θ_max_ = 30.5°, θ_min_ = 1.9°
*T*_min_ = 0.591, *T*_max_ = 0.746	*h* = −10→10
31084 measured reflections	*k* = −12→12
4288 independent reflections	*l* = −15→15

### Refinement

**Table:**

Refinement on *F*^2^	0 restraints
Least-squares matrix: full	Hydrogen site location: inferred from neighbouring sites
*R*[*F*^2^ > 2σ(*F*^2^)] = 0.035	H-atom parameters constrained
*wR*(*F*^2^) = 0.089	*w* = 1/[σ^2^(*F*_o_^2^) + (0.0395*P*)^2^ + 0.2342*P*] where *P* = (*F*_o_^2^ + 2*F*_c_^2^)/3
*S* = 1.02	(Δ/σ)_max_ = 0.002
4288 reflections	Δρ_max_ = 0.57 e Å^−^^3^
181 parameters	Δρ_min_ = −0.47 e Å^−^^3^

### Special details

**Table:**

Geometry. All esds (except the esd in the dihedral angle between two l.s. planes) are estimated using the full covariance matrix. The cell esds are taken into account individually in the estimation of esds in distances, angles and torsion angles; correlations between esds in cell parameters are only used when they are defined by crystal symmetry. An approximate (isotropic) treatment of cell esds is used for estimating esds involving l.s. planes.

### Fractional atomic coordinates and isotropic or equivalent isotropic displacement parameters (Å^2^)

**Table:**

	*x*	*y*	*z*	*U*_iso_*/*U*_eq_	
Br1	0.17874 (3)	1.01452 (2)	0.68946 (2)	0.05997 (10)	
Cl1	0.27559 (10)	−0.20635 (8)	0.05226 (6)	0.07320 (19)	
N1	0.1843 (2)	0.4932 (2)	0.37610 (15)	0.0451 (4)	
N2	0.3231 (2)	0.36057 (19)	0.53108 (14)	0.0408 (3)	
N3	0.3389 (2)	0.57048 (19)	0.69517 (14)	0.0416 (3)	
C1	0.3258 (3)	0.0867 (2)	0.36107 (18)	0.0460 (4)	
H1	0.3669	0.0842	0.4441	0.055*	
C2	0.3317 (3)	−0.0449 (3)	0.27905 (19)	0.0502 (5)	
H2	0.3753	−0.1360	0.3063	0.060*	
C3	0.2715 (3)	−0.0391 (3)	0.15560 (19)	0.0506 (5)	
C4	0.2054 (3)	0.0941 (3)	0.11321 (19)	0.0538 (5)	
H4	0.1664	0.0962	0.0299	0.065*	
C5	0.1979 (3)	0.2241 (3)	0.19575 (19)	0.0493 (5)	
H5	0.1515	0.3137	0.1678	0.059*	
C6	0.2590 (3)	0.2232 (2)	0.32120 (17)	0.0420 (4)	
C7	0.2538 (2)	0.3616 (2)	0.40937 (17)	0.0400 (4)	
C8	0.2942 (2)	0.5038 (2)	0.57750 (16)	0.0390 (4)	
C9	0.2078 (3)	0.5896 (2)	0.48503 (17)	0.0408 (4)	
C10	0.1670 (3)	0.7408 (2)	0.51508 (19)	0.0453 (4)	
H10	0.1095	0.7982	0.4567	0.054*	
C11	0.2162 (3)	0.8041 (2)	0.63802 (19)	0.0444 (4)	
C12	0.3010 (3)	0.7204 (2)	0.72501 (18)	0.0452 (4)	
H12	0.3328	0.7673	0.8056	0.054*	
C13	0.4300 (3)	0.4809 (3)	0.78916 (18)	0.0489 (5)	
H13A	0.5192	0.5528	0.8446	0.059*	
H13B	0.4916	0.4010	0.7491	0.059*	
C14	0.3080 (3)	0.4023 (3)	0.8615 (2)	0.0550 (5)	
H14	0.3600	0.3461	0.9228	0.066*	
C15	0.1374 (4)	0.4035 (3)	0.8484 (3)	0.0709 (7)	
H15A	0.0789	0.4579	0.7884	0.085*	
H15B	0.0727	0.3499	0.8990	0.085*	

### Atomic displacement parameters (Å^2^)

**Table:**

	*U*^11^	*U*^22^	*U*^33^	*U*^12^	*U*^13^	*U*^23^
Br1	0.05960 (16)	0.03754 (12)	0.08491 (19)	0.00970 (9)	0.01673 (12)	0.00593 (10)
Cl1	0.0863 (5)	0.0732 (4)	0.0594 (3)	0.0184 (3)	0.0175 (3)	−0.0136 (3)
N1	0.0482 (9)	0.0466 (9)	0.0414 (8)	0.0074 (7)	0.0058 (7)	0.0105 (7)
N2	0.0446 (9)	0.0398 (8)	0.0399 (8)	0.0079 (7)	0.0079 (7)	0.0090 (6)
N3	0.0486 (9)	0.0397 (8)	0.0383 (8)	0.0076 (7)	0.0078 (7)	0.0100 (6)
C1	0.0462 (11)	0.0510 (11)	0.0425 (10)	0.0094 (9)	0.0090 (8)	0.0066 (8)
C2	0.0508 (12)	0.0515 (12)	0.0511 (11)	0.0131 (9)	0.0133 (9)	0.0050 (9)
C3	0.0479 (11)	0.0554 (12)	0.0489 (11)	0.0053 (9)	0.0158 (9)	−0.0042 (9)
C4	0.0542 (12)	0.0648 (14)	0.0407 (10)	0.0030 (10)	0.0055 (9)	0.0041 (9)
C5	0.0487 (11)	0.0521 (12)	0.0464 (10)	0.0054 (9)	0.0033 (9)	0.0092 (9)
C6	0.0370 (9)	0.0468 (11)	0.0426 (9)	0.0016 (8)	0.0086 (8)	0.0058 (8)
C7	0.0369 (9)	0.0427 (10)	0.0419 (9)	0.0040 (8)	0.0082 (7)	0.0094 (8)
C8	0.0390 (9)	0.0395 (9)	0.0410 (9)	0.0042 (7)	0.0095 (7)	0.0117 (7)
C9	0.0394 (10)	0.0421 (10)	0.0441 (9)	0.0056 (8)	0.0096 (8)	0.0145 (8)
C10	0.0441 (10)	0.0407 (10)	0.0546 (11)	0.0079 (8)	0.0094 (9)	0.0182 (9)
C11	0.0436 (10)	0.0347 (9)	0.0584 (11)	0.0048 (8)	0.0161 (9)	0.0094 (8)
C12	0.0508 (11)	0.0403 (10)	0.0458 (10)	0.0038 (8)	0.0130 (9)	0.0054 (8)
C13	0.0532 (12)	0.0523 (12)	0.0416 (10)	0.0121 (9)	0.0019 (9)	0.0107 (9)
C14	0.0662 (15)	0.0521 (12)	0.0472 (11)	0.0093 (10)	0.0024 (10)	0.0170 (9)
C15	0.0673 (17)	0.0755 (18)	0.0765 (17)	0.0097 (13)	0.0144 (13)	0.0348 (14)

### Geometric parameters (Å, º)

**Table:**

Br1—C11	1.886 (2)	C6—C1	1.395 (3)
Cl1—C3	1.744 (2)	C6—C5	1.401 (3)
N1—C7	1.342 (3)	C7—C6	1.464 (3)
N1—C9	1.371 (3)	C8—C9	1.431 (3)
N2—C7	1.375 (2)	C9—C10	1.373 (3)
N2—C8	1.327 (2)	C10—H10	0.9300
N3—C8	1.352 (2)	C11—C10	1.399 (3)
N3—C12	1.355 (3)	C11—C12	1.373 (3)
N3—C13	1.477 (2)	C12—H12	0.9300
C1—H1	0.9300	C13—H13A	0.9700
C1—C2	1.381 (3)	C13—H13B	0.9700
C2—H2	0.9300	C13—C14	1.480 (3)
C2—C3	1.384 (3)	C14—H14	0.9300
C4—H4	0.9300	C14—C15	1.287 (4)
C4—C3	1.378 (3)	C15—H15A	0.9300
C5—H5	0.9300	C15—H15B	0.9300
C5—C4	1.377 (3)		
			
Br1···C14^i^	3.624 (2)	N3···H15A	2.5350
Br1···C15^i^	3.660 (3)	C1···C11^ii^	3.532 (3)
Br1···C2^ii^	3.677 (2)	C1···C12^ii^	3.472 (3)
Br1···C6^iii^	3.729 (2)	C5···C13^ii^	3.595 (3)
Br1···H10^iv^	3.1682	C6···C12^ii^	3.470 (3)
Cl1···H12^v^	2.8304	C7···C8^ii^	3.512 (3)
Cl1···H14^vi^	3.1002	C7···C10^iii^	3.499 (3)
Cl1···H15B^vii^	2.9750	C8···C15	3.559 (4)
N3···C6^ii^	3.437 (3)	C9···C9^iii^	3.473 (3)
N1···H5	2.6087	C12···C15	3.378 (3)
N1···H15A^iii^	2.5889	C5···H13A^ii^	2.8677
N2···H13B	2.5360	C12···H15A	2.8971
N2···H1	2.5251	H12···H13A	2.4427
			
C7—N1—C9	102.65 (16)	N2—C8—C9	111.53 (16)
C8—N2—C7	101.17 (15)	N3—C8—C9	120.81 (17)
C8—N3—C12	119.16 (16)	N1—C9—C8	107.12 (17)
C8—N3—C13	120.08 (16)	N1—C9—C10	132.60 (18)
C12—N3—C13	120.76 (16)	C10—C9—C8	120.27 (18)
C6—C1—H1	119.5	C9—C10—C11	116.59 (18)
C2—C1—C6	120.95 (19)	C9—C10—H10	121.7
C2—C1—H1	119.5	C11—C10—H10	121.7
C1—C2—H2	120.5	C10—C11—Br1	120.71 (15)
C1—C2—C3	118.9 (2)	C12—C11—Br1	117.03 (16)
C3—C2—H2	120.5	C12—C11—C10	122.18 (18)
C4—C3—Cl1	119.49 (17)	N3—C12—C11	120.99 (18)
C4—C3—C2	121.6 (2)	N3—C12—H12	119.5
C2—C3—Cl1	118.93 (18)	C11—C12—H12	119.5
C5—C4—H4	120.4	N3—C13—H13A	108.8
C5—C4—C3	119.2 (2)	N3—C13—H13B	108.8
C3—C4—H4	120.4	N3—C13—C14	113.74 (18)
C6—C5—H5	119.5	H13A—C13—H13B	107.7
C4—C5—C6	120.9 (2)	C14—C13—H13A	108.8
C4—C5—H5	119.5	C14—C13—H13B	108.8
C1—C6—C7	120.23 (17)	C13—C14—H14	116.6
C1—C6—C5	118.48 (19)	C15—C14—C13	126.8 (2)
C5—C6—C7	121.28 (18)	C15—C14—H14	116.6
N2—C7—C6	120.09 (17)	C14—C15—H15A	120.0
N1—C7—N2	117.53 (17)	C14—C15—H15B	120.0
N1—C7—C6	122.39 (17)	H15A—C15—H15B	120.0
N2—C8—N3	127.66 (17)		
			
C9—N1—C7—N2	−0.3 (2)	C6—C5—C4—C3	1.1 (3)
C9—N1—C7—C6	−179.75 (17)	C5—C6—C1—C2	0.0 (3)
C7—N1—C9—C8	0.5 (2)	C7—C6—C1—C2	−179.96 (19)
C7—N1—C9—C10	179.6 (2)	C1—C6—C5—C4	−0.8 (3)
C8—N2—C7—N1	0.0 (2)	C7—C6—C5—C4	179.11 (19)
C8—N2—C7—C6	179.45 (17)	N1—C7—C6—C1	−177.28 (18)
C7—N2—C8—N3	−178.58 (19)	N1—C7—C6—C5	2.8 (3)
C7—N2—C8—C9	0.3 (2)	N2—C7—C6—C1	3.3 (3)
C12—N3—C8—N2	178.81 (19)	N2—C7—C6—C5	−176.66 (18)
C12—N3—C8—C9	0.0 (3)	N2—C8—C9—N1	−0.5 (2)
C13—N3—C8—N2	−0.5 (3)	N2—C8—C9—C10	−179.82 (17)
C13—N3—C8—C9	−179.29 (18)	N3—C8—C9—N1	178.47 (17)
C8—N3—C12—C11	0.8 (3)	N3—C8—C9—C10	−0.8 (3)
C13—N3—C12—C11	−179.93 (19)	N1—C9—C10—C11	−178.3 (2)
C8—N3—C13—C14	−97.3 (2)	C8—C9—C10—C11	0.8 (3)
C12—N3—C13—C14	83.4 (2)	C12—C11—C10—C9	0.0 (3)
C6—C1—C2—C3	0.6 (3)	Br1—C11—C10—C9	176.73 (14)
C1—C2—C3—Cl1	−178.98 (17)	Br1—C11—C12—N3	−177.67 (15)
C1—C2—C3—C4	−0.4 (3)	C10—C11—C12—N3	−0.8 (3)
C5—C4—C3—Cl1	178.14 (17)	N3—C13—C14—C15	1.2 (4)
C5—C4—C3—C2	−0.5 (3)		

Symmetry codes: (i) *x*, *y*+1, *z*; (ii) −*x*+1, −*y*+1, −*z*+1; (iii) −*x*, −*y*+1, −*z*+1; (iv) −*x*, −*y*+2, −*z*+1; (v) *x*, *y*−1, *z*−1; (vi) −*x*+1, −*y*, −*z*+1; (vii) −*x*, −*y*, −*z*+1.

### Hydrogen-bond geometry (Å, º)

**Table:**

*D*—H···*A*	*D*—H	H···*A*	*D*···*A*	*D*—H···*A*
C15—H15*A*···N1^iii^	0.93	2.59	3.454 (4)	155

Symmetry code: (iii) −*x*, −*y*+1, −*z*+1.
